# Identification of potential hub genes and drugs in septic kidney injury: a bioinformatic analysis with preliminary experimental validation

**DOI:** 10.3389/fmed.2025.1502189

**Published:** 2025-03-17

**Authors:** Shujun Sun, Yuanyuan Ding, Dong Yang, Jiwei Shen, Tianhao Zhang, Guobin Song, Xiangdong Chen, Yun Lin, Rui Chen

**Affiliations:** ^1^Department of Anesthesiology, Union Hospital, Tongji Medical College, Huazhong University of Science and Technology, Wuhan, China; ^2^Institute of Anesthesia and Critical Care Medicine, Union Hospital, Tongji Medical College, Huazhong University of Science and Technology, Wuhan, China; ^3^Key Laboratory of Anesthesiology and Resuscitation (Huazhong University of Science and Technology), Ministry of Education, Wuhan, China; ^4^Department of Pain, Union Hospital, Tongji Medical College, Huazhong University of Science and Technology, Wuhan, China; ^5^Department of Anesthesiology, Zhejiang Hospital, Hangzhou, China

**Keywords:** sepsis-associated kidney injury, text mining, drug discovery, mRNA-miRNA co-expression networks, immune cell infiltration

## Abstract

**Background:**

Sepsis-associated kidney injury (SAKI) is a prevalent complication in intensive care unit (ICU) patients with sepsis. Diagnosis currently relies on clinical assessment, urine output, and serum creatinine levels, yet effective clinical treatments remain scarce. Our objectives are to explore prospective, targeted medications for the treatment of septic kidney injury and to employ bioinformatics to identify key genes and pathways that may be implicated in the pathogenesis of SAKI.

**Methods:**

We utilized the GEO database for differential gene screening. Related genes of septic kidney injury were identified through Pubmed2Ensembl, followed by annotation and visualization of gene ontology biological processes and KEGG pathways using DAVID. Protein–protein interactions were analyzed with the STRING database, and hub genes were identified using Cytoscape software. Candidate genes were further validated through Metascape. The CTD database was employed to uncover the relationship between hub genes and acute kidney injury (AKI). CIBERSORT was applied to evaluate the infiltration of immune cells and their association with hub genes. Hub genes were experimentally verified through qPCR detection. Lastly, the Drug–Gene Interaction Database (DGIdb) was utilized to identify drug–gene interactions.

**Results:**

Six genes, including TNF, CXCL8, IL-6, IL-1β, IL-2, and IL-10, were associated with three major signaling pathways: the COVID-19 adverse outcome pathway, an overview of pro-inflammatory and pro-fibrotic mediators, and the interleukin-10 signaling pathway. Additionally, 12 targeted drugs were identified as potential therapeutic agents.

## Introduction

Sepsis-associated acute kidney injury (SAKI) is a common complication of sepsis, accounting for 26 to 50% of all acute kidney injuries (AKI) (1). It is characterized by a high mortality rate, poor prognosis, and poses a significant threat to patient survival. AKI is an independent risk factor for increased mortality in sepsis patients, with the mortality rate of SAKI patients reaching up to 70% (2). Even if patients survive, the risk of developing residual chronic kidney disease (CKD) is significantly increased, imposing a substantial economic burden on society and patients’ families. In clinical practice, especially for patients with AKI in the ICU, it is crucial to have biomarkers that can predict disease progression and severity.

Recent studies have primarily discovered biomarkers through wet experiments, with a notable lack of bioinformatics methods. However, emerging research indicates that certain drug ingredients can be used to treat SAKI, as they can influence related pathways involved in SAKI. For example, flavonoid fisetin has been shown to alleviate kidney inflammation in SAKI mouse models (3). A previous study identified NOX4 as a potential therapeutic target in SAKI (4). However, these study relied solely on wet experiments without employing bioinformatics methods, which may limit the comprehensiveness of the findings. Additionally, several miRNAs are implicated in the pathogenesis of SAKI and are expected to serve as potential therapeutic targets (5). Exploring the connections between SAKI targets and related miRNAs could help identify key genes involved in the condition. Despite these findings, the currently recommended treatment strategy for SAKI remains primarily symptomatic and supportive, with no specific treatments available for SAKI patients. The etiology of SAKI is complex and not yet fully understood, involving multiple factors. A deeper understanding of the pathogenesis of SAKI is essential to identify new therapeutic targets and strategies. Therefore, identifying key genes of SAKI and searching for effective biomarkers are crucial for early diagnosis, prevention, and intervention of SAKI.

Text mining of biomedical literature is a valuable technique for generating novel hypotheses, as it can reveal previously unknown associations between genes and diseases (6). Given the exponential growth of information that surpasses manual management capabilities, text mining techniques have become essential in life sciences. Particularly, they are instrumental in electronic drug discovery processes, including drug target identification and pharmacogenomics (7). By integrating text mining with other analytical technologies, researchers can identify new gene candidates and potential applications for existing medications. The use of Gene Ontology (GO) cell signaling pathway maps aids in pinpointing target genes or regulators that are amenable to targeting. This is achieved by assessing the connections among a set of genes within a network of protein interactions, which can help in prioritizing genes for further study. This approach highlights that genes with high connectivity tend to cluster together within the network (8). Once the target genes are identified, drug–gene interaction analysis is conducted to develop potential drug candidates (9).

Our study aims to identify potential genes and medications for the treatment of SAKI by leveraging text mining, functional and signaling pathway analysis, and database analytic techniques. Our approach began with a comprehensive analysis of publicly available gene expression datasets related to SAKI. Initially, we compiled a preliminary list of relevant genes to explore potential therapeutic agents for septic kidney injury. Subsequently, we generated a list of high-priority target genes by integrating extensive data on these genes through functional and signaling network enrichment analysis. This was followed by the use of protein–protein interaction networks to further refine the gene selection. Finally, the examination of drug–gene interaction data led to the identification of potential drug candidates. Our findings provide a foundation for future research endeavors and may offer a basis for the development of new targeted therapeutic approaches as potential treatments for SAKI.

## Methods and materials

### Data sources

A gene expression profile associated with septic kidney injury (GSE94717) was obtained from the Gene Expression Omnibus (GEO) database, which is publicly accessible at http://www.ncbi.nih.gov/geo. This dataset can be freely downloaded by esearchers and the general public for further analysis. Microarray platform GSE242059 miRNA expression profile for GPL16791 (Affymetrix Human Gene 1.0 ST Array), which includes human kidney tissue samples. The data can be downloaded online by the general audience. GSE232404 was obtained from the GEO database, used for immune cell infiltration analysis.

### Identification of differentially expressed genes

The gene expression dataset GSE94717, related to SAKI, was downloaded from the GEO database.^1^ The data was analyzed online using GEO2R, where it was divided into SAKI and sepsis-non AKI and normal groups. The criteria for identifying differentially expressed genes (DEGs) were set with a *p*-value less than 0.05 and a log-fold change greater than 1 or less than −1.

### Text mining

In this research, we employed pubmed2ensembl^2^ for text mining purposes. We specified “*Homo sapiens*” as the target species and utilized the search term “septic kidney injury.” The process involved selecting options such as “Search PubMed ID,” “Search up to 100,000 document IDs,” and applying the “MEDLINE PubMedID filter” to compile a comprehensive gene list.

### GO biological process and KEGG pathway analyses

DAVID (Database for Annotation, Visualization, and Integrated Discovery)^3^ is a powerful resource that facilitates the transition from raw data collection to the extraction of biological insights, particularly for genome-scale datasets (10). This integrated approach has proven advantageous in interpreting high-throughput experimental data. In our study, the genes identified through text mining were imported into DAVID for further analysis. We utilized various annotation categories, including KEGG pathway, GO Molecular Function, GO Cellular Component, and GO Biological Process, to analyze the gene set with “*Homo sapiens*” specified as the organism of interest. To adjust the *p*-values and control for multiple testing, we employed the “hypergeometric” statistical test and applied the false discovery rate (FDR) correction method.

### Protein–protein interactions

The STRING database^4^ aims to compile a comprehensive map of all known and predicted protein interactions, encompassing both functional and physical associations (11). In our analysis, we utilized the STRING database to explore the network of protein–protein interactions for the genes identified in the previous phase. We selected “multiple proteins” from the menu bar on the left and specified “*Homo sapiens*” as the organism of interest. We set the confidence level to medium, corresponding to a score of 0.400, to ensure a balance between the number of interactions and their reliability. This approach allowed us to construct a network that visualizes the complex interactions among the target genes.

Following the construction of the protein–protein interaction network, we proceeded to analyze and visualize this network using Cytoscape software. Cytoscape is a powerful tool that provides a visual representation of the integration between genotype, biological networks, and gene expression data (12). To identify core functional modules within the network, we employed the Molecular Complex Detection (MCODE) plugin within Cytoscape (13). This plugin facilitated the identification of densely connected regions within the network, which are often indicative of biologically significant interactions. Through this process, we were able to pinpoint the hub genes that play central roles in the network.

### Functional enrichment of hub genes

Metascape^5^ is a web-based portal designed to offer experimental biologists a comprehensive resource for annotating and analyzing gene lists. This platform integrates over 40 distinct knowledge sources into a single gateway, combining feature-rich interactome analysis, gene annotation, and member search capabilities. Metascape also facilitates the comparison of datasets from multiple, diverse experiments, enhancing the ease of data interpretation. The platform’s rapid, one-click analysis interface generates interpretable outputs, significantly streamlining the user experience (14). In our analysis, we set the cutoff value at a *p*-value of less than 0.05 to identify significant results.

### Prediction of the target miRNAs of the DEGs

To predict the target miRNAs of the identified hub genes, we utilized five reputable online miRNA databases: miRWalk, miRDB, TargetScan, DIANA-micro, and miRcode. Subsequently, we constructed a visual mRNA-miRNA co-expression network using Cytoscape software, which visualized the interactions between the mRNAs and miRNAs. For a miRNA to be considered a target, it had to be listed in at least four of the consulted databases.

### The association between screened hub genes and AKI

The Comparative Toxicogenomics Database (CTD)^6^ is a valuable resource that provides comprehensive information on the relationships between chemicals, genes, and diseases (15). In our study, we utilized the CTD database to explore the association between the identified hub genes and the risk of AKI.

### Immune infiltration analysis

The CIBERSORT package was employed to estimate the immune cell composition within the samples. This algorithm utilizes gene expression arrays in conjunction with predefined immune feature matrices to calculate the relative proportions of 22 distinct immune cell subpopulations. The dataset used is GSE232404. To compare the differences in immune cell infiltration between patients with SAKI and normal samples, we used the “immuneconv” in the R package to perform this analysis and utilized the ggplot2 package in the R programming language for data visualization.

### Quantitative real-time PCR

Total RNA was extracted from SAKI mouse (established using cecal ligation and puncture) (16) kidney tissue using the RNeasy Mini Kit (QIAGEN) according to the manufacturer’s protocol. The quantity and purity of RNA were assessed using a NanoDrop 2000 spectrophotometer (Thermo Fisher Scientific) and by calculating the 260/280 nm absorbance ratio. Only samples with a ratio between 1.8 and 2.1 were used for subsequent analysis. The reverse transcription of RNA to complementary DNA (cDNA) was performed using the High-Capacity cDNA Reverse Transcription Kit (Applied Biosystems) in a 20 μL reaction volume, containing 1 μg of total RNA, according to the supplier’s instructions. The reaction was incubated at 25°C for 10 min, followed by 37°C for 120 min, and finally heated to 85°C for 5 min to inactivate the enzyme. Quantitative real-time PCR was conducted using the StepOnePlus Real-Time PCR System (Applied Biosystems) with gene-specific primers and the PowerUp SYBR Green Master Mix (Thermo Fisher Scientific). Each reaction contained 2 μL of cDNA, 10 μL of 2× PowerUp SYBR Green Master Mix, 0.5 μM of each primer, and nuclease-free water to a final volume of 20 μL. The thermal cycling conditions were as follows: an initial denaturation at 95°C for 2 min, followed by 40 cycles of 95°C for 15 s and 60°C for 1 min. A melting curve analysis was performed at the end of each PCR to confirm the specificity of the amplification. The relative expression levels of target genes were calculated using the $2^{-\mathrm{\Delta \Delta CT}}$ method, normalizing to the endogenous control gene, β-actin, which exhibited stable expression across all samples. Each sample was analysed once with three independent biological replicates per group to ensure stability and reproducibility of results, and non-template controls were included in each run to monitor potential contamination.

### Drug–gene interactions

The Drug Gene Interaction Database (DGIdb)^7^ is a comprehensive resource that consolidates information on drug–gene interactions from multiple sources (17). In our analysis, we applied the following specific filters to ensure the reliability and relevance of the identified drug–gene interactions: ① “Remove NA from interaction types” to exclude undefined interaction types, ② “Set the query score value to >4” to ensure high confidence in the gene-drug associations, ③ “Set the interaction score as >2” to prioritize strong interactions. We then imported the hub genes identified from our Cytoscape analysis into DGIdb. Through this process, we were able to identify specific medications with potential therapeutic effects for septic kidney injury.

### Statistical analysis

We conducted statistical analyses using the stats package in the R programming language (version 4.1.0). To compare continuous variables between groups, we employed Student’s *t*-test. The results are presented as the mean ± standard deviation (SD) or standard error of the mean (S.E.M.). Statistical significance was determined at a threshold of *p*-values less than 0.05, with the following notation for significance levels: ^***^p < 0.001, ^**^p < 0.01, and ^*^p < 0.05.

## Results

### Identification of differentially expressed genes

**Figure 1:**
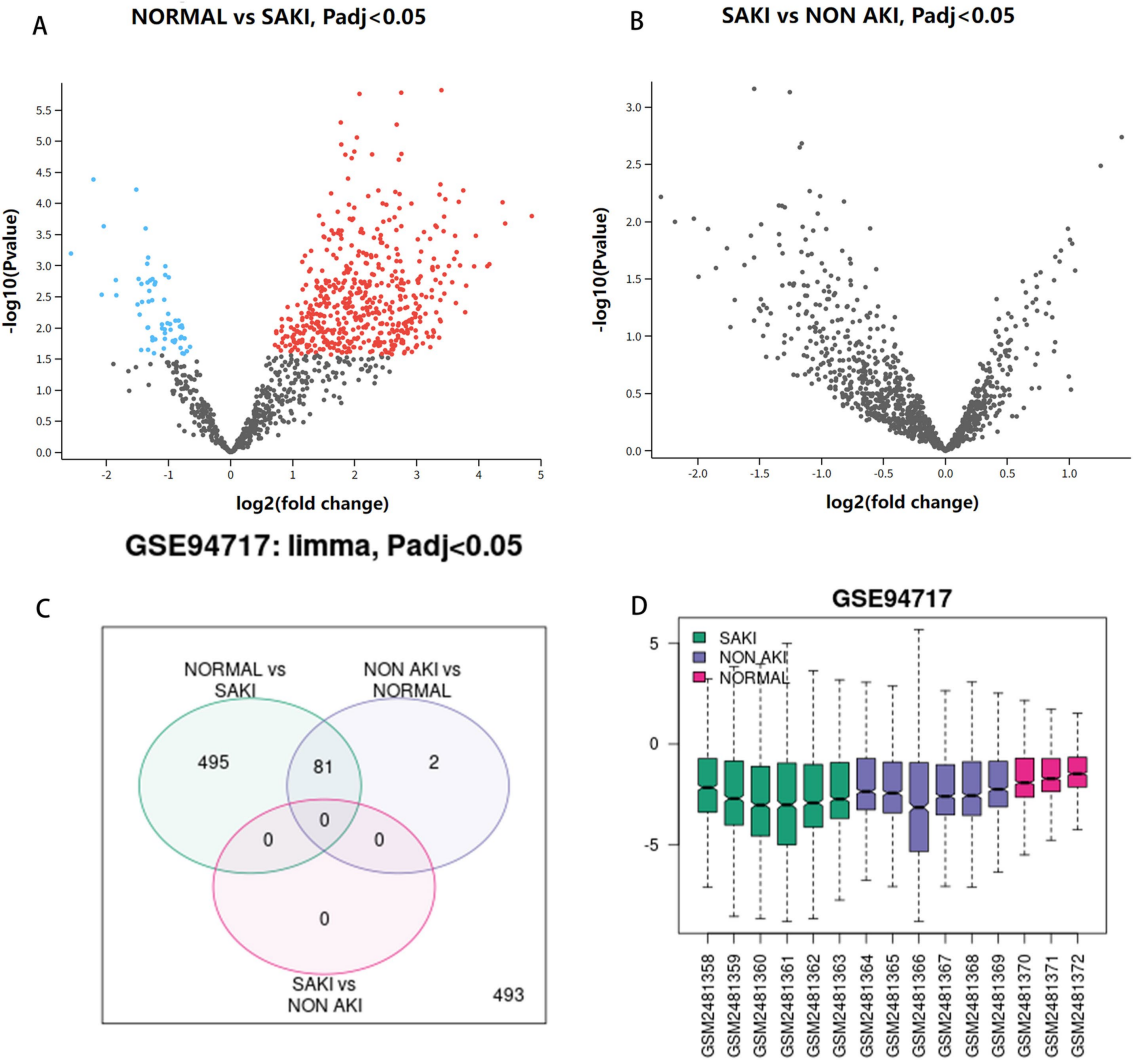
Analysis of DEGs. **(A)** Volcanic maps of normal group and SAKI group. **(B)** Volcanic maps of SAKI group and sepsis not AKI group. **(C)** Venn diagram. **(D)** Box plot.

We began by normalizing the Sepsis Kidney Injury (SKI) dataset, GSE94717, which consists of six sepsis kidney injury tissue samples, six sepsis non-kidney injury tissue samples, and three healthy tissue samples. The identification of DEGs was based on criteria of “*p* < 0.05 and a log-fold change greater than 1 or less than −1.” Utilizing GEO2R, we performed differential gene expression analysis on the GSE94717 dataset to identify DEGs between the SAKI group and the non SAKI and normal group. The volcano plot of multiple comparisons shows that there are upregulated and downregulated differentially expressed genes between healthy and SAKI populations, but unfortunately, there are no statistically significant differentially expressed genes between the non SAKI and SAKI groups. The subsequent circle chart shows 81 differentially expressed genes. Box plot displays detailed information of GSM sequence as shown in Figure 1.

### Results of text mining

Using pubmed2ensembl, we searched the entire database, then culminating in a refined list of 71 genes, which are detailed in Table 1.

**Table 1:** These 71 genes were related to septic kidney injury.

Results of text mining
IL-10, REN, SELP, CRP, S100A6, PHGDH, SST, GC, F3, CEP70, IL-1β, IL1R1, UROD, IL-18, LEP, SLC17A5, NOVA2, NOS1, G6PD, SERPINE1, MPO, LPO, GSTA1, AK1, COIL, EPO, LRSAM1, PAH, AMBP, NKRF, NPHS1, TSC22D3, GPT, PIK3C2A, THBD, CDKN2A, CST3, EDN1, PRL, ICAM1, AGT, APC, MB, RAPGEF5, IL-6, CPQ, PPP3CA, GAS6, AQP2, NOS2, C16orf82, IL-2, TNF, ENO1, POMC, GUSB, PTH, CXCL8, RETN, ALB, RRBP1, B2M, GGT1, PLAT, GTPBP4, AVP, SERPINF2, TJP, ELANE, PRTN3, VWF

### Results of GO biological process and KEGG pathway analyses

**Figure 2:**
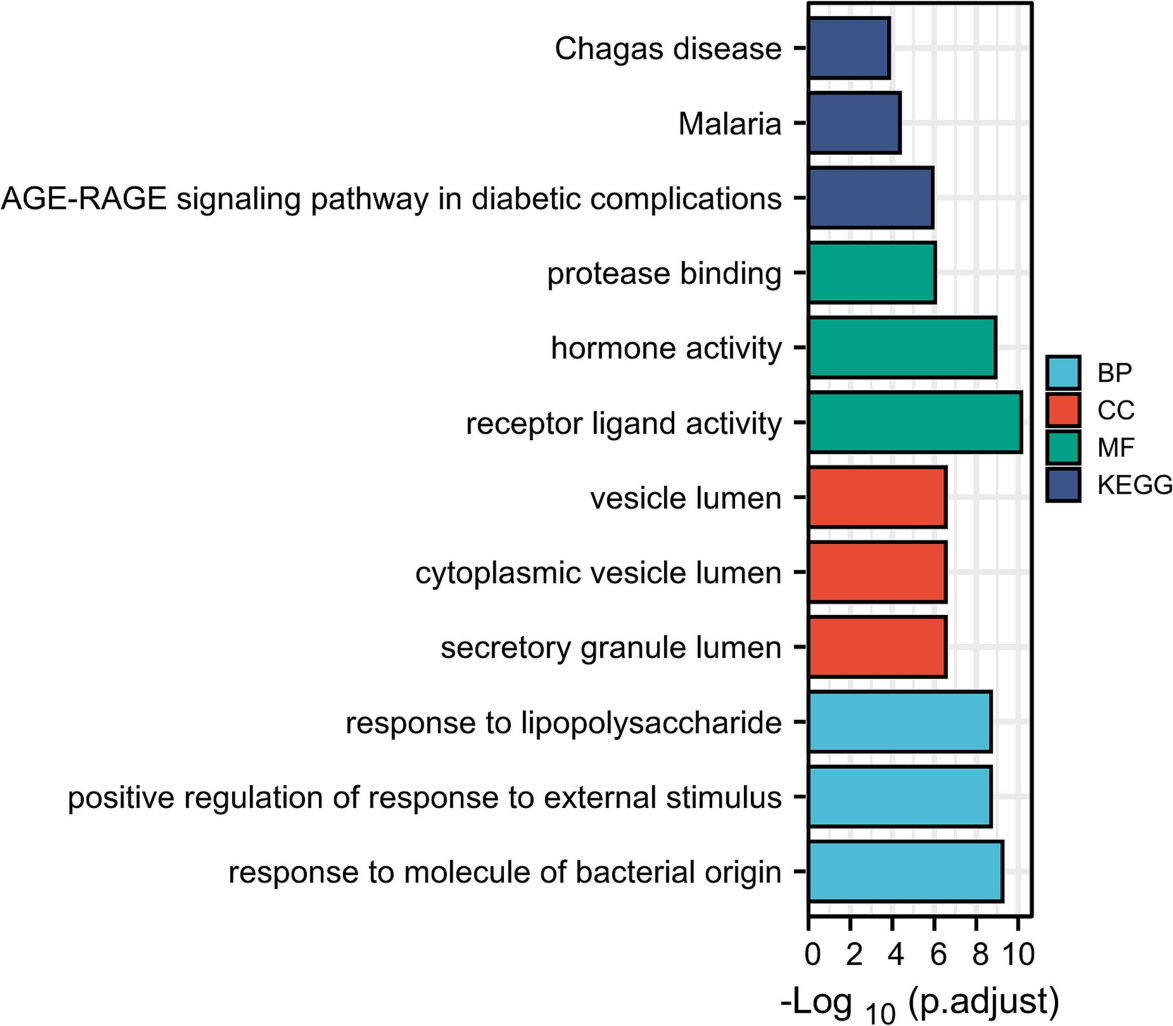
Enrichment analysis of genes in SAKI (David). GO_biological processes, GO_cellular component category, GO_molecular function analysis and KEGG pathway analysis.

The GO biological processes of the 71 genes were analyzed using DAVID, revealing terms that were highly enriched and closely related to the pathology of renal injury in sepsis. To ensure that the annotations selected were most relevant to the pathology of renal injury in sepsis, we applied a stringent corrected cut-off value of *p* = 0.01. The five most enriched biological process annotations included: response to extracellular space (*p*_adj = 2.69 × 10^−23^), extracellular region (*p*_adj = 9.73 × 10^−15^), lipopolysaccharide (*p*_adj = 5.45 × 10^−11^), hormone activity (*p*_adj = 1.73 × 10^−10^), extracellular exosome (*p*_adj = 2.25 × 10^−9^). Other significantly enriched biological processes included positive regulation of interleukin-8 production, regulation of insulin secretion, response to activity, negative regulation of lipid storage, positive regulation of neuroinflammatory response, positive regulation of tyrosine phosphorylation of STAT protein, and positive regulation of T-helper 1 cell cytokine production. These findings are visualized in Figure 2 and detailed in Table 2.

**Table 2:** Significantly enriched GO terms and KEGG pathways of genes.

Category	Term	Description	Count	*p*-value
BP	GO:0002237	Response to molecule of bacterial origin	15	2.22 × 10^−13^
BP	GO:0032103	Positive regulation of response to external stimulus	14	1.76 × 10^−12^
BP	GO:0032496	Response to lipopolysaccharide	14	2.35 × 10^−12^
CC	GO:0034774	Secretory granule lumen	11	3.64 × 10^−9^
CC	GO:0060205	Cytoplasmic vesicle lumen	11	6.22 × 10^−9^
CC	GO:0031983	Vesicle lumen	11	6.41 × 10^−9^
MF	GO:0048018	Receptor ligand activity	17	3.36 × 10^−13^
MF	GO:0005179	Hormone activity	10	1.12 × 10^−11^
MF	GO:0002020	Protease binding	8	1.28 × 10^−8^
KEGG	hsa04933	AGE-RAGE signaling pathway in diabetic complications	9	6.48 × 10^−9^
KEGG	hsa05144	Malaria	6	4.72 × 10^−7^
KEGG	hsa05142	Chagas disease	7	2.32 × 10^−6^

During the KEGG pathway enrichment analysis, we set the adjusted *p*-value (*p*val_adj) cutoff at 0.05 to identify significantly enriched pathways. The top five most significantly enriched pathways included: AGE-RAGE signaling pathway in diabetic complications (*p*_adj = 1.82 × 10^−7^), malaria (*p*_adj = 1.82 × 10^−7^), African trypanosomiasis (*p*_adj = 1.17 × 10^−5^), Chagas disease (*p*_adj = 2.59 × 10^−5^), rheumatoid arthritis (*p*_adj = 1.39 × 10^−4^). These pathways are visualized in Figure 2 and detailed in Table 2.

### Results of protein–protein interactions

**Figure 3:**
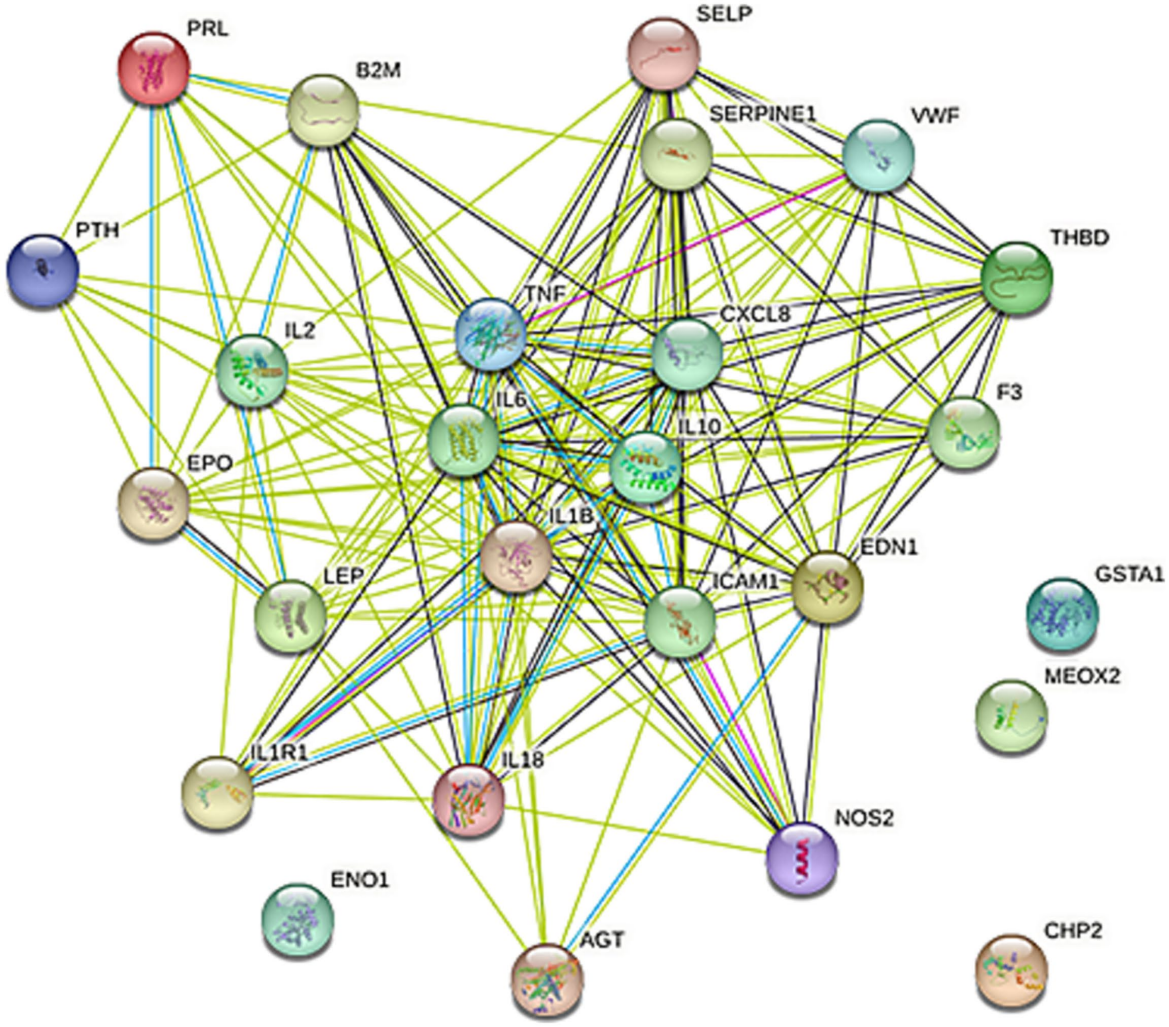
PPI network. The protein–protein interaction network of the 28 targeted genes was produced using STRING with a medium confidence score of 0.400. Connecting line color indicates the types of interaction evidence, with the confidence score set at 90%.

**Figure 4:**
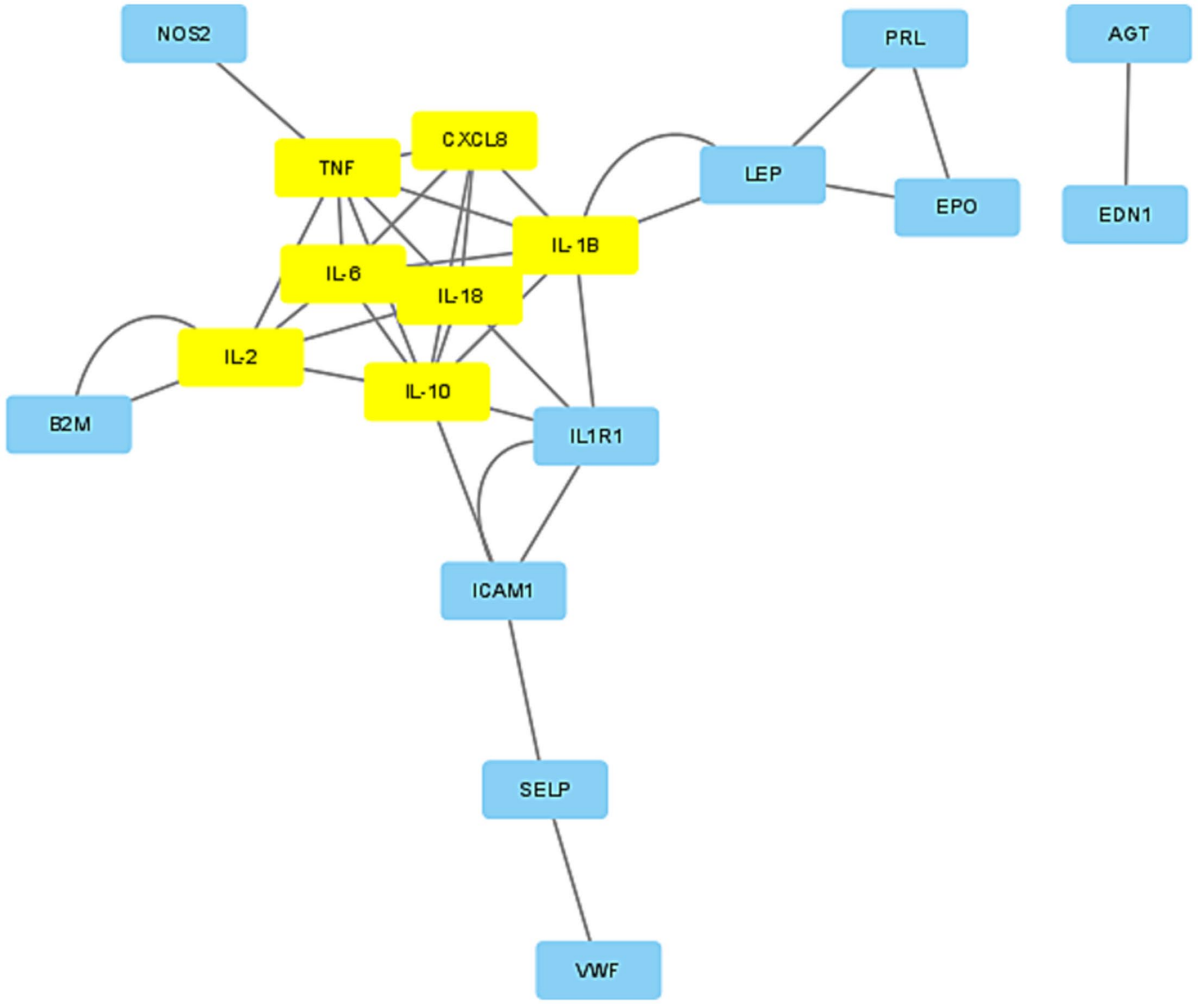
Gene module. Significant gene module (K-core 4). The yellow ones are the hub genes.

The protein–protein interaction network for the 28 target genes was analyzed using the STRING database, resulting in the identification of 24 associated genes, as depicted in Figure 3. The interaction data was exported from STRING in “.tsv” (tab-separated values) format and imported into Cytoscape. We applied the “MCODE” application in Cytoscape to configure a K-Core of 4, using the default settings for all other parameters. MCODE was also utilized to filter out two significant modules from the network. Module 1 was particularly noteworthy, comprising seven genes: TNF, CXCL8, IL-6, IL-1β, IL-18, IL-2, and IL-10, as shown in Figure 4. Consequently, these seven genes from Module 1 were selected for further research (see Table 3).

**Table 3:** The details of module 1 genes.

Gene symbol	Full name	Function
IL-2	Interleukin-2	A cytokine in the immune system, facilitating various aspects of immune response. An pro-inflammatory cytokine
IL-10	Interleukin-10	A cytokine with pleiotropic effects in immune regulation and inflammation. An anti-inflammatory cytokine
IL-6	Interleukin-6	A versatile cytokine that plays a crucial role in immune regulation, inflammation, acute phase response, hematopoiesis, metabolism, and chronic inflammatory diseases. An pro-inflammatory cytokine
IL-18	Interleukin-18	A cytokine involved in the regulation of immune responses. An pro-inflammatory cytokine
IL-1β	Interleukin-1β	A cytokine in the immune system that mediates a wide range of inflammatory responses. An pro-inflammatory cytokine
TNF	Tumor necrosis factor	A central cytokine in the immune system that regulates inflammation, induces apoptosis, stimulates the acute phase response, and has various effects on immune cells and blood vessels. An pro-inflammatory cytokine
CXCL8	C-X-C motif chemokine ligand 8	A key chemokine in the immune system, primarily responsible for the recruitment and activation of neutrophils

### Function analysis of hub genes

**Figure 5:**
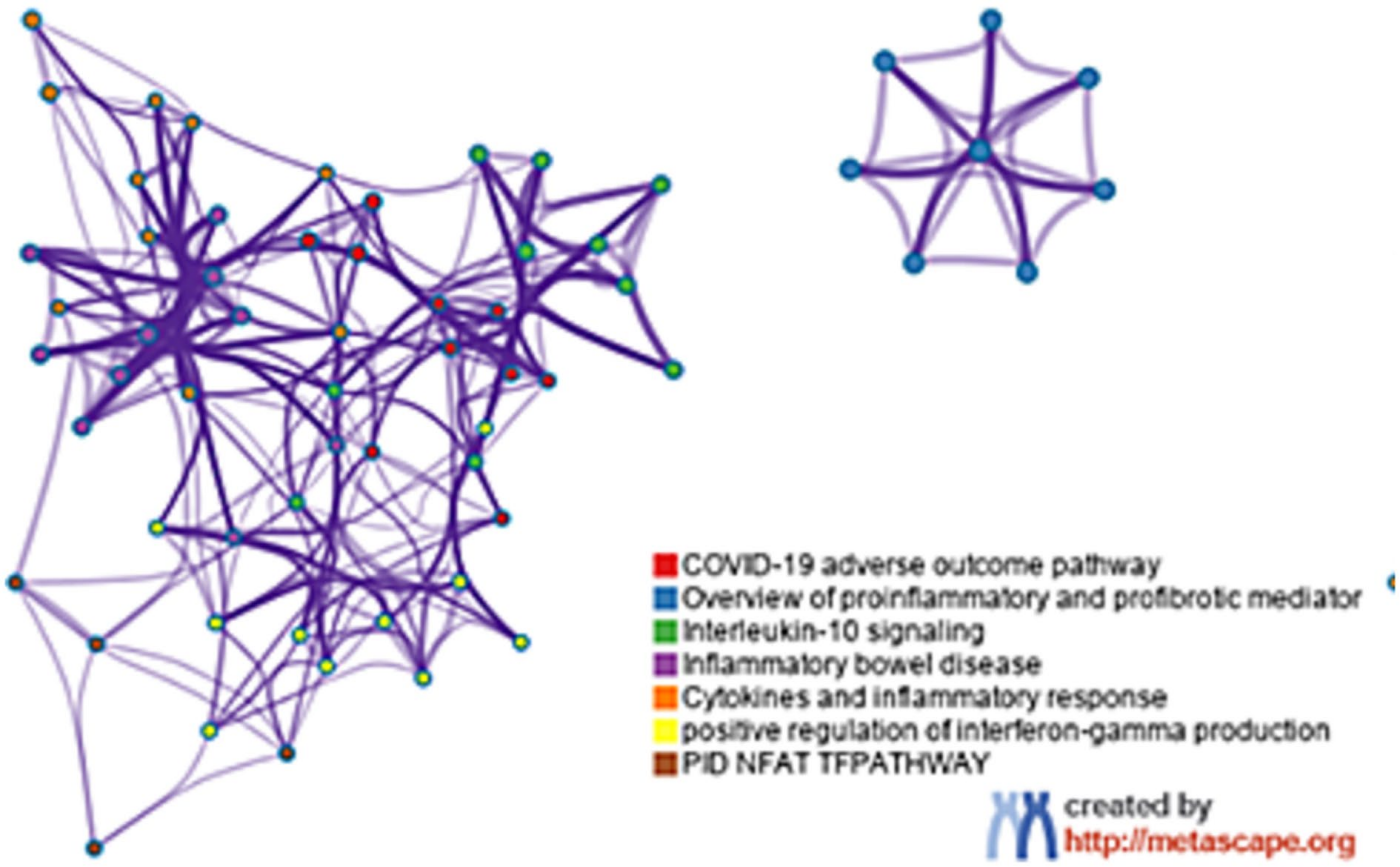
The function analysis of hub genes (*p* < 0.05). The functions of hub genes were mainly enriched in COVID-19 adverse outcome pathway, overview of pro-inflammatory and pro-fibrotic mediator and interieukin-10 signaling.

The functional annotation obtained from Metascape indicates that the core gene primarily influences several critical pathways, including the COVID-19 unfavorable outcome pathway, an overview of pro-inflammatory and pro-fibrotic mediators, and interleukin-10 signaling, as illustrated in Figure 5.

### Construction of mRNA-miRNA co-expression networks

**Figure 6:**
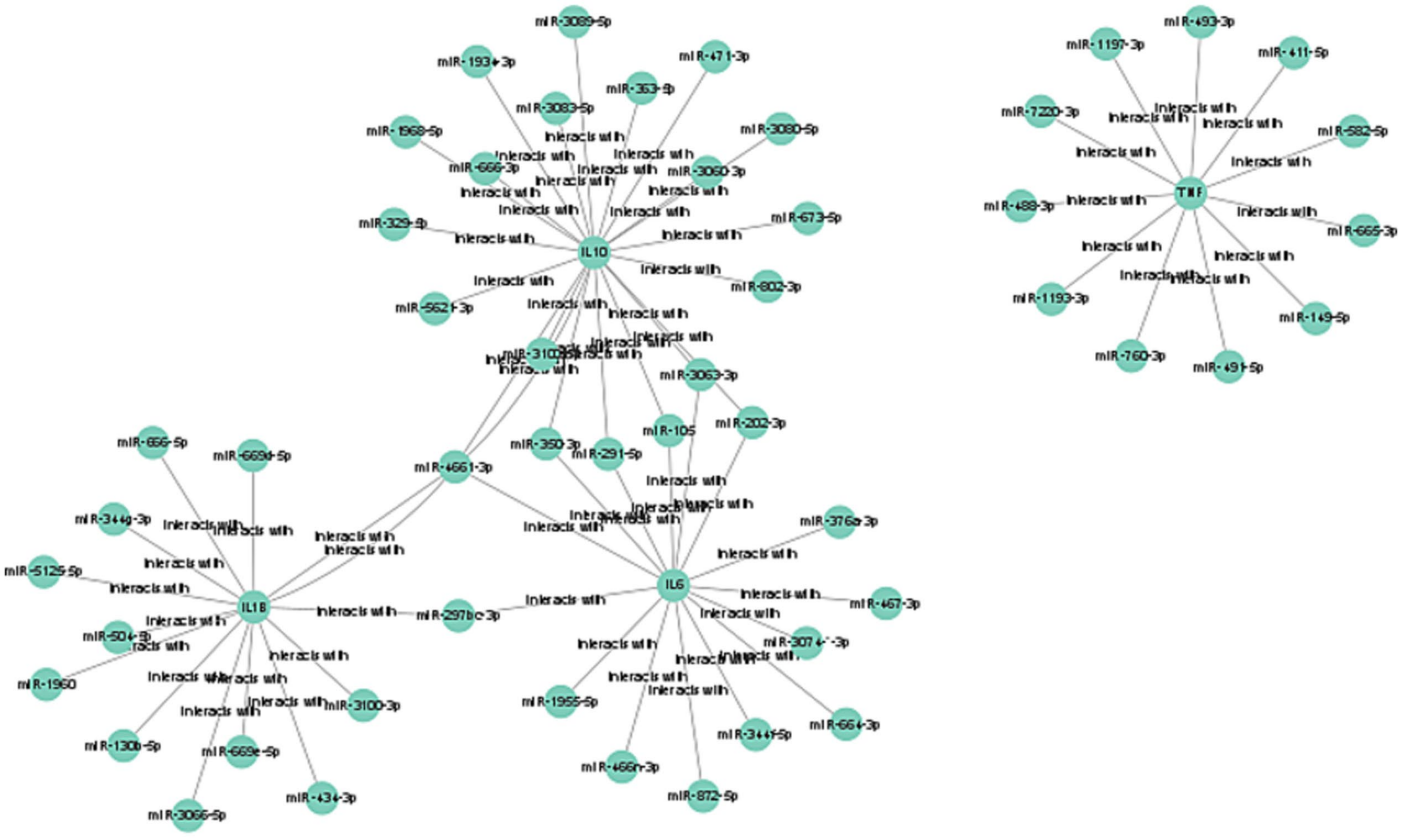
Construction of mRNA-miRNA co-expression networks. The mRNA-miRNA co-expressed network was constructed by Cytoscape.

MicroRNAs (miRNAs) are known to regulate gene expression by binding to the 5′ or 3′ untranslated regions (UTRs) of target mRNAs, playing a significant role in the development of septic kidney injury. Through the prediction of four target miRNA clusters that specifically express hub genes, we utilized five online miRNA databases and identified 56 target miRNAs and 62 mRNA-miRNA pairs. Subsequently, we constructed a co-expression network between miRNA and mRNA using Cytoscape, which is visualized in Figure 6.

### The interaction between hub genes and AKI based on the CTD database

**Figure 7:**
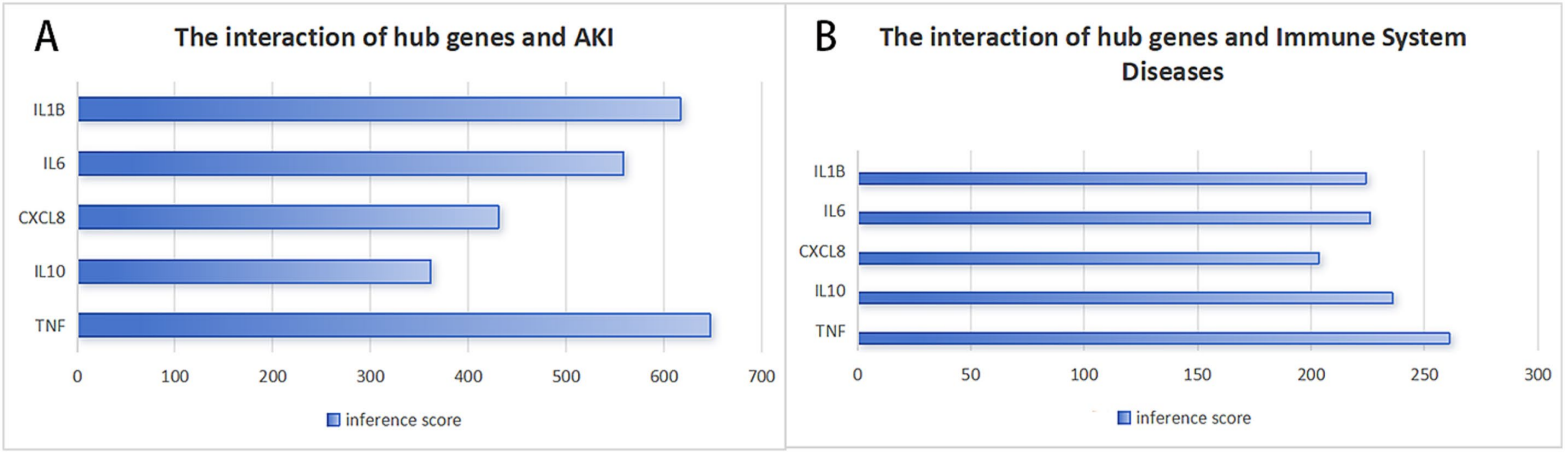
Recognization of potential crucial genes related to AKI by CTD database. **(A)** The interaction of hub genes and AKI, **(B)** The interaction of hub genes and immune system diseases.

We utilized the CTD to assess the association between selected central genes and AKI, aiming to identify genes that may play a significant role in AKI. Figure 7 illustrates that there are five hub genes that target both AKI and immune system diseases. Among these, TNF has the highest inference score, suggesting the strongest relationship with AKI and a pivotal role in the immune system. Additionally, IL-1β and IL-6, which are typical pro-inflammatory cytokines, score relatively high and are crucial in regulating inflammatory responses. While CXCL8 and IL-10 have lower scores, their contributions to AKI and the immune system are still considerable; CXCL8 is primarily involved in neutrophil recruitment, and IL-10 functions as an anti-inflammatory cytokine.

### Immune cell infiltration in SAKI

**Figure 8:**
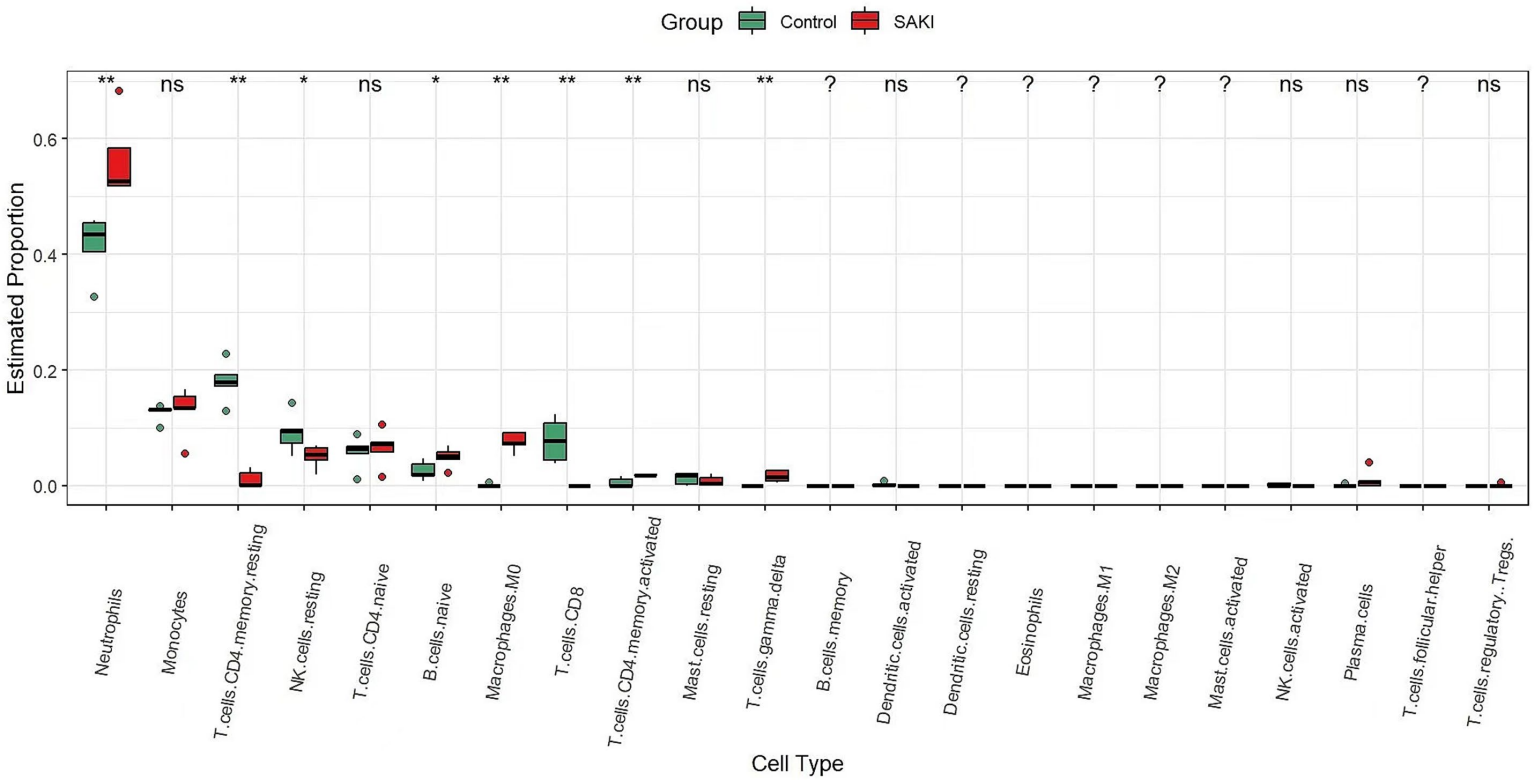
The difference in infiltrating immune cells between SAKI and the normal group. The SAKI group is represented in red, while the normal group is represented in green. (ns: no statistical difference,* < 0.05, ** < 0.01, and *** < 0.001).

Numerous studies have established a strong correlation between renal tissue injury, patient prognosis, and outcomes with the presence and activity of immune cells. Utilizing the CIBERSORT algorithm, we analyzed the distribution of 22 immune cell subtypes in renal tissues. As depicted in the box plot of Figure 8, there is an increased presence of neutrophils, B cells, M0 macrophages, CD4 T cell memory activated and γδ T cells in the glomerular tissue of SAKI patients. Conversely, a decreased presence of CD4 T cell memory resting, NK cells, and CD8 T cells was observed. Furthermore, the genes CXCL8, IL-1β, TNF, IL-6, and IL-10 were found to be significantly upregulated, suggesting their involvement in key biological processes and immune responses associated with SAKI.

### Quantitative real-time PCR of differentially expressed genes

**Figure 9:**
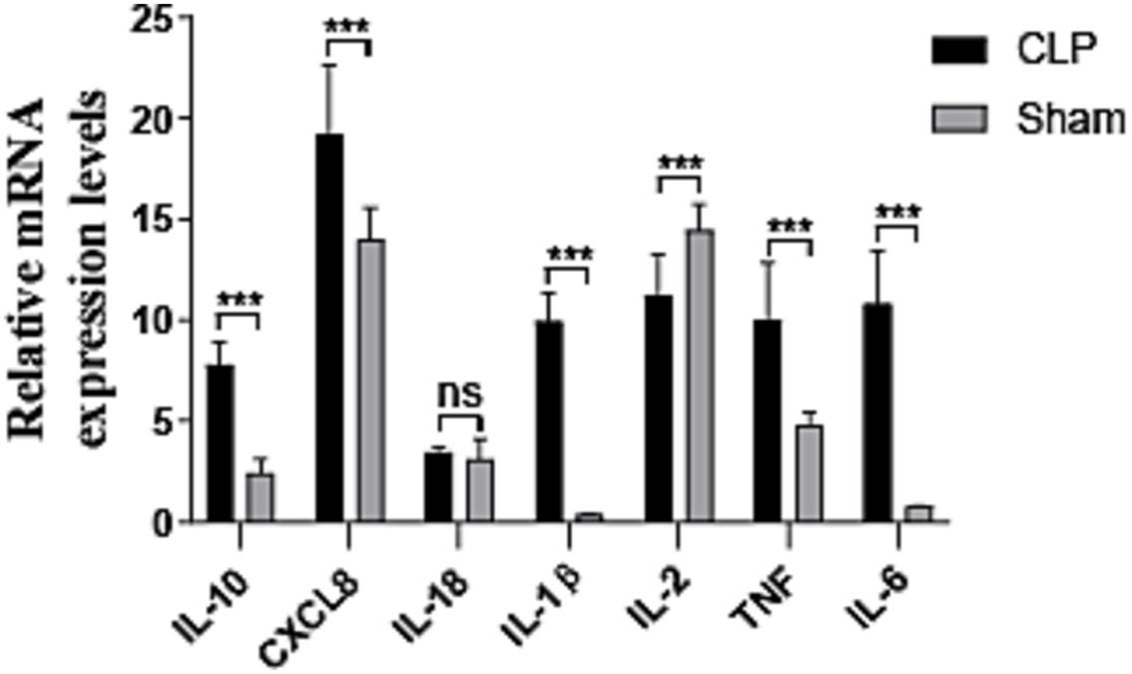
Results of quantitative real-time PCR experiments for the hub seven genes (*n* = 3). CLP, SAKI group; sham, control group; ns: no statistical difference, * < 0.05, ** < 0.01, and *** < 0.001.

We compared the identified genes and assessed their expression levels using real-time fluorescence qPCR, as shown in Figure 9. In the CLP-induced sepsis kidney injury model, the IL-2 gene exhibited low expression, whereas the CXCL8, IL-1β, TNF, IL-6, and IL-10 genes demonstrated high expression levels. The expression levels of IL-18 did not show statistical significance.

### Results of drug–gene interactions

The drug–gene interactions of TNF, CXCL8, IL-6, IL-1β, IL-2 and IL-10 were analyzed using the DGIdb. After reviewing the literature, we identified 12 candidate medications with therapeutic potential for septic kidney injury, which are listed in Table 4.

**Table 4:** Candidate drugs targeting genes for septic kidney injury.

Number	Drug	Gene	Query score	Interaction score
1	Golimumab	TNF	21.52	4.55
2	Placulumab	TNF	12.91	2.73
3	ABX-IL8	CXCL8	8.61	2.21
4	Siltuximab	IL-6	17.22	9.89
5	Olokizumab	IL-6	17.22	9.89
6	Clazakizumab	IL-6	12.91	7.42
7	PF-04236921	IL-6	8.61	2.47
8	Sirukumab	IL-6	4.3	2.47
9	Elsilimomab	IL-6	4.3	2.47
10	Canakinumab	IL-1β	25.82	10.03
11	Rilonacept	IL-1β	8.61	3.34
12	SCH-708980	IL-10	4.3	3.64

## Discussion

SAKI is one of the earliest and most common comorbidities in patients with severe sepsis. Elevated serum creatinine levels or reduced urine output are diagnostic criteria for septic kidney injuries, irrespective of the underlying cause or subsequent complications. While these diagnostic techniques are beneficial, they have limitations that underscore the necessity for enhanced strategies to treat septic kidney damage (18). However, research on pharmacological interventions as adjunctive treatments for SAKI remains limited. Our study identified six key genes associated with SAKI: TNF, CXCL8, IL-6, IL-1β, IL-2, and IL-10. These genes are involved in critical signaling pathways, including the COVID-19 adverse outcome pathway, pro-inflammatory and pro-fibrotic mediator pathways, and the IL-10 signaling pathway. Known to play a key role in the pathogenesis of SAKI, these genes are expected to serve as potential therapeutic targets. The potential drug candidates identified in this study, such as TNF inhibitors (e.g., golimumab and placulumab), IL-6 inhibitors (e.g., siltuximab, olokizumab, clazakizumab, PF-04236921, sirukumab, and ELSILIMOMAB), IL-1β inhibitors (e.g., canakinumab and rilonacept), and an IL-10 inhibitor (SCH-708980), offer promising avenues for targeted therapies. These drugs have shown efficacy in treating other inflammatory conditions and could be repurposed for the treatment of SAKI. For instance, TNF inhibitors have been successfully used in treating rheumatoid arthritis and other inflammatory diseases, suggesting their potential for mitigating the inflammatory response in SAKI (19). However, clinical translation requires validation in trials and addressing SAKI’s complex pathophysiology. Our findings lay a foundation for future research and targeted therapies, emphasizing the need for biomarker development and combination treatments.

The GO annotation analysis of DEGs indicates that these genes are primarily enriched in collagen-containing extracellular matrix, cytokine production regulation, and immune response processes. These findings are highly consistent with the characteristics of septic kidney injury, such as the deposition of circulating immune complexes, induction of glomerular cell proliferation, excessive production of extracellular matrix (ECM), secretion of inflammatory cytokines, and infiltration of multiple immune cells (20). Additionally, the KEGG pathway analysis revealed that pathways related to malaria, and rheumatoid arthritis were significantly enriched, further confirming the important role of immune response and inflammation in the occurrence and development of septic kidney injury. Subsequently, a protein–protein interaction (PPI) network was constructed to identify six hub genes, namely TNF, CXCL8, IL-6, IL-1β, IL-2, and IL-10. The enrichment analysis of these hub genes demonstrated that they are highly correlated with the adverse outcome pathway of COVID-19, the overview of pro-inflammatory and pro-fibrotic mediators, and the interleukin-10 signaling pathway. These results highlight the potential involvement of these hub genes in the pathogenesis of septic kidney injury and suggest their potential as therapeutic targets.

Patients with COVID-19 exhibit a spectrum of disease severity, from those with mild upper respiratory symptoms or asymptomatic to those with severe lung injury requiring hospitalization, and potentially progressing to hyperinflammation and acute respiratory distress syndrome (ARDS) (21, 22). During a COVID-19 infection, viral particles infect adjacent uninfected cells as they traverse the respiratory system, triggering a cytokine storm that initiates a robust immunological response. This cascade can lead to alterations in immune cells, particularly lymphocytes, causing the immune system to become dysregulated (23). Consequently, the reduction in circulating lymphocytes may serve as a diagnostic biomarker for the severity of SARS-CoV-2 infection (24). Prior research has indicated that pro-inflammatory cytokines such as IL-1β, IL-6, IL-12, IFNγ, IL-10, and MCP1, as well as cytokines like TNFα, IL-15, and IL-17, are associated with lung injury and inflammation in SARS-CoV and MERS-CoV infections, respectively. Notably, elevated levels of cytokines such as MCP1, IP10, IFNγ, and IL-1β can elicit Th1 responses in T cells, and the severity of the illness may be linked to this cytokine storm (25). Kidney damage is one of the many downstream consequences, primarily caused by inflammatory dysregulation, with the likelihood of direct kidney injury increasing in proportion to the severity of the inflammatory response. The inflammatory cytokines generated along the COVID-19 adverse outcome pathway may be associated with septic kidney injury linked to this pathway. By examining the target genes IL-1β, IL-10, IL-6, and TNF, we can confirm that their primary biological function is to regulate inflammation, thereby influencing SAKI. These target genes are also involved in the COVID-19 adverse outcome pathway. Our analysis, building on previous studies, further confirms that IL-1β, IL-10, IL-6, and TNF are key inflammatory cytokines associated with this pathway, suggesting that targeting these cytokines with specific drugs may provide novel therapeutic strategies for SAKI.

Renal fibrosis, characterized by the accumulation of scarring within the renal parenchyma, represents the common end-stage in the progression of most CKD. In the context of CKD, the uncontrolled deposition of fibrotic matrix leads to the destruction of the kidney’s structural integrity, reduction in blood supply, and impairment of organ function. This fibrotic process diminishes the tissue’s capacity for repair, ultimately culminating in kidney failure (26). Signaling pathways that are essential for kidney development, such as the Wnt, Hedgehog (Hh), and Notch pathways, also play a pivotal role in renal fibrosis. For instance, the renal tubule-specific ablation of β-catenin has been shown to exacerbate the severity of AKI (27). In CKD, renal tubular cells are subjected to a sustained activation of the Wnt/β-catenin pathway due to the upregulation of various Wnt ligands (28). This prolonged activation can result in mesenchymal fibrosis and epithelial dedifferentiation, driven by the continuous stimulation of epithelial β-catenin (29). Fibroblasts and peri-mesenchymal cells are additional key targets of Wnt ligands, underscoring the critical role of Wnt ligands in paracrine signaling between mesenchymal myofibroblasts and damaged epithelial cells. Notably, Wnt1, released by renal tubules, is sufficient to induce interstitial fibrosis independently (30).

Our data indicate that the target genes IL-1β, IL-6, CXCL8, and TNF are implicated in SAKI. These genes are integral components of the pro-inflammatory and pro-fibrotic mediator signaling pathway, which has been previously implicated in kidney damage. IL-1β is a crucial cytokine in the immune system that plays a significant role in inflammatory responses, particularly in SAKI. The mechanisms of action of IL-1β in SAKI include the following five aspects: activation of the inflammatory response, damage to renal endothelial function, promotion of tubular injury, induction of immune response dysregulation, and impact on renal hemodynamics (31). Research has shown that IL-1β levels are typically elevated in sepsis patients, and its levels are positively correlated with the severity of SAKI. Experimental studies have demonstrated that inhibiting IL-1β function or blocking its receptors can provide renal protection, reducing the incidence of SAKI. IL-6 also plays an important role in SAKI, primarily by regulating the inflammatory response, affecting renal microcirculation, and promoting tubular injury, thereby exacerbating renal failure (32). Excessive activation of IL-6 is closely associated with immune dysfunction and kidney damage in sepsis. Laboratory studies have shown that inhibiting IL-6 production or its signaling pathway can alleviate the occurrence and progression of SAKI. TNF is a key pro-inflammatory cytokine in sepsis and is significantly involved in SAKI by inducing inflammatory responses, endothelial damage, and tubular cell injury (33). TNF not only exacerbates kidney damage by enhancing the immune response but may also worsen sepsis progression by promoting immunosuppression. CXCL8, as a key pro-inflammatory chemokine, plays an important role in sepsis-related acute kidney injury. By promoting the chemotaxis and activation of neutrophils, CXCL8 exacerbates renal inflammation and microcirculatory disorders, leading to tubular injury and functional failure (34). IL-1β, IL-6, and TNF are established as pro-inflammatory cytokines, while the expression of CXCL8 is linked to epithelial β-catenin (35, 36). Our qPCR analysis reveals that the relative mRNA expression level of CXCL8 is significantly elevated in the cecal ligation and puncture (CLP) group compared to the sham-operated group. CXCL8 is instrumental in the recruitment and activation of neutrophils, which are pivotal in inflammation and fibrosis (37). The pro-inflammatory cytokines IL-1β, IL-6, and TNF are also key players in this process. Our study, in conjunction with existing research, demonstrates that these target genes can modulate inflammation and fibrosis through their influence on pro-inflammatory and pro-fibrotic mediators during kidney injury. Our findings corroborate the association between these biological processes and the genes IL-1β, IL-6, CXCL8, and TNF, suggesting that a potential therapeutic strategy may involve the identification of drugs that specifically target these cytokines to mitigate SAKI.

IL-10 is a cytokine with potent anti-inflammatory properties that plays a crucial role in infection control by suppressing the immune system’s response to infections, thereby protecting the host from excessive immune-mediated damage (38). IL-10 is expressed by a variety of immune cells, including B cells, TReg cells, CD8^+^ T cells, and subsets of TH1, TH2, and TH17 cells (39–41). Additionally, innate immune system cells such as mast cells, eosinophils, neutrophils, natural killer cells (NK), dendritic cells (DC), and macrophages also produce IL-10. IL-10 suppresses the proliferation of TH1-type responses by acting on DCs and macrophages (42, 43), but it has the opposite effect on TH2 cells and allergic reactions. Furthermore, IL-10 stimulates mast cells and enhances the function of B, NK, and CD8^+^ T cells (44). Toll-like receptor 2 (TLR2) agonists promote the production of IL-10 through antigen-presenting cells (APCs) (45, 46). Following TLR ligation, both pro-inflammatory cytokines and IL-10 are produced via Toll/IL-1 receptor (TIR) domain-containing adapter molecules, such as myeloid differentiation primary response protein 88 (MYD88) and TIR domain-containing adapter protein-inducible IFNβ (TRIF; also known as TICAM1) (47, 48). IL-10 also activates a critical survival pathway composed of PI3K and its downstream substrates AKT/PKB and P70S6K. Moreover, IL-10 inhibits the activation of the p38/MAPK (mitogen-activated protein kinase) pathway, which is necessary for the translation of TNF.

The IL-10 signaling pathway includes the p38/MAPK pathway and the PI3K-AKT signaling pathway (49). Research has indicated that the IL-10 signaling pathway is involved in and influences SAKI. Concurrently, IL-10 can affect SAKI, suggesting that IL-10 participates in and leads to the impact of these two signaling pathways on the anti-inflammatory response, thereby affecting the condition of SAKI. This demonstrates that IL-10 target genes can influence the occurrence of SAKI through the IL-10 signaling pathway. The potent anti-inflammatory properties of IL-10 can be harnessed to treat acute inflammatory diseases related to sepsis.

To date, no research has definitively confirmed the correlation between IL-2 and SAKI. The role of IL-2 in sepsis is complex and dualistic. In the early stages of sepsis, IL-2 enhances T cell proliferation and activation, promotes the immune response, and aids in clearing infections. However, an excessive immune response may lead to renal immune damage, exacerbate inflammation, and result in renal failure. During the immunosuppressive phase of sepsis, a deficiency in IL-2 may worsen immune suppression and increase the risk of secondary infections. Therefore, the role of IL-2 in SAKI needs to be comprehensively considered for its dual impact on the immune system and carefully evaluated in clinical treatment.

The CTD database results showed that TNF, IL-10, IL-1β, and IL-6 exhibited high scores in AKI, reflecting the close relationship between these key genes and the occurrence and development of AKI. Given that IL-6, TNF, and IL-10 were found to be implicated in this pathway in our investigation, a potential treatment strategy could involve identifying medications that specifically target these cytokines. This approach aligns with the understanding that targeting IL-10 and related cytokines may offer new therapeutic avenues for managing SAKI, as suggested by the research and clinical observations summarized in the provided sources.

TNF-targeting medications, such as placulumab and golimumab, are monoclonal antibodies designed to target and neutralize TNF, thereby reducing inflammation. Golimumab, approved for conditions including rheumatoid arthritis, psoriatic arthritis, ulcerative colitis, non-radiographic axial spondyloarthritis, ankylosing spondylitis, and juvenile idiopathic arthritis, exemplifies the clinical utility of such agents (50). In contrast, placulumab remains in the realm of scientific investigation and is not yet clinically available. Our study suggests that TNF-targeting drugs may hold potential for the treatment of SAKI, as inflammation is a known exacerbating factor in such cases.

IL-6 inhibitors, including siltuximab, olokizumab, clazakizumab, PF-04236921, sirukumab, and elsilimomab, represent another class of therapeutics with demonstrated efficacy in conditions like Castleman disease and rheumatoid arthritis (51–55). Our research indicates that these IL-6 inhibitors could also be therapeutically beneficial in SAKI, given their established anti-inflammatory effects. IL-1β inhibitors, such as rilonacept and canakinumab, have shown promise in reducing lung cancer incidence and mortality, as well as in the treatment of autoinflammatory relapsing fever syndrome and Still’s disease (56). Our study supports the notion that these inhibitors could positively influence the treatment of SAKI by modulating inflammatory responses. SCH-708980, an IL-10 inhibitor, is currently in scientific research phases. While it has the potential to enhance immune responses and aid in clearing infections, which could benefit SAKI, its use may also intensify inflammatory responses, necessitating cautious consideration in treatment strategies. MicroRNAs (miRNAs), small non-coding RNAs that regulate protein production by interacting with mRNAs, have been implicated in the pathogenesis of SAKI (57, 58). Our study identified miR-105, miR-3063-3p, and miR-297bc-3p as potential therapeutic targets for SAKI. However, the roles and mechanisms of other miRNAs in SAKI require further investigation to elucidate their relationship with the pathophysiology of the condition and to inform clinical treatment strategies.

It is important to acknowledge the limitations of this study. Firstly, the sample size is inadequate. While our study has identified several potential hub genes and drug–gene interactions through robust bioinformatics approaches, the relatively small sample size used in our analyses may limit the generalizability of our findings. The dataset GSE94717, which we utilized for differential gene expression analysis, included a limited number of samples (six SAKI samples, six sepsis samples, and three healthy controls). This small sample size may affect the statistical power of our analyses and the ability to detect subtle differences or interactions among genes and drug targets. Secondly, additional basic and clinical research is essential to substantiate the findings and explore the underlying molecular mechanisms. Although bioinformatics analysis can provide valuable information for target screening, these targets still need to be validated in the laboratory. Common validation methods include: gene knockout or overexpression: knocking out or overexpressing candidate genes through mouse models, cell lines, or CRISPR technology to study their effects on disease models. Gene editing technology: utilizing CRISPR-Cas9 technology to validate the function of targets, such as studying the role of the gene or protein in diseases. RNA interference and antibody technology: using siRNA, shRNA, or antibodies to inhibit targets and observe their therapeutic effects in cell or animal models. In our study only the target genes were detected using PCR and we recognise that additional experimental validation, such as protein expression analysis or functional studies, would help to confirm the role of these genes in septic kidney injury, but given the funding constraints we were unable to complete these experiments at this stage. Therefore, we urge caution in interpreting our results and suggest that further experimental studies are needed to fully validate the functional significance of the identified genes. Thirdly, the genetic data used in CIBERSORT analysis may not fully capture the complexities of phenotypic plasticity, heterotypic cell–cell interactions, and disease-induced interference, potentially leading to overestimation or underestimation of certain immune cell types, despite the method’s relatively low estimation bias compared to other approaches. Fourth, there are limitations in translating our findings to clinical applications and validating the proposed drug candidates. Although potential target genes and drugs were identified through bioinformatics analysis, these findings have not yet been validated in clinical trials, leaving their efficacy and safety in actual treatments uncertain. Additionally, the process of translating drugs from the laboratory to clinical application is complex and time-consuming, requiring multiple phases of clinical trials and regulatory approvals. Therefore, further research and validation are necessary to confirm the clinical applicability and therapeutic potential of these drug candidates. Finally, it should be noted that the database used for human samples in our study was derived from peripheral blood, whereas the mouse experiments analyzed renal tissues. There may be differences in immune cell composition and function between these two types of samples. While our analyses provide valuable insights into the molecular mechanisms of septic kidney injury, the differences in tissue sources may affect the direct comparability and extrapolation of our findings. Future studies are needed to bridge the gap between blood and kidney tissue findings and to ensure that insights derived from one tissue type can be reliably applied to the other.

## Conclusion

In the present study, we proposed a methodology to identify potential genes and pharmacological agents associated with septic kidney injury. Our investigation led to the identification of 12 potential medications targeting six genes, the majority of which have not been explored in the context of septic kidney injury. These findings provide a foundation for future research endeavors and may pave the way for the development of novel targeted therapeutics as potential treatments for septic kidney damage.

## Data Availability

The original contributions presented in the study are included in the article/supplementary material, further inquiries can be directed to the corresponding authors.

## Footnotes

^1^ https://www.ncbi.nlm.nih.gov/geo

^2^ http://pubmed2ensembl.ls.manchester.ac.uk/

^3^ http://david.ncifcrf.gov

^4^ http://stringdb.org

^5^ https://metascape.org

^6^ http://www.ctdbase.org

^7^ http://www.dgidb.org
